# Filovirus Surveillance in Communities Bordering Equatorial Guinea, Marburg Outbreak, Cameroon, 2023

**DOI:** 10.3201/eid3208.260117

**Published:** 2026-08

**Authors:** Jill-Léa Ramassamy, Flaubert Auguste Mba Djondzo, Innocent Ndong Bass, Ginette Edoul, Dowbiss Meta-Djomsi, Nadine Lamare, Maeliss Champagne, Célestin Godwe, Kono Léon, Cavour Tadjouteu, Audrey Lacroix, Nicole Vidal, Guillaume Thaurignac, Elisabeth Dibongue, Eric Delaporte, Martine Peeters, Ahidjo Ayouba, Charles Kouanfack

**Affiliations:** Université de Montpellier, IRD, INSERM, Montpellier, France (J.-L. Ramassamy, M. Champagne, A. Lacroix, N. Vidal, G. Thaurignac, E. Delaporte, M. Peeters, A. Ayouba); Centre de Recherche sur les Maladies Emergentes et Réémergentes (CREMER), Yaoundé, Cameroon (F.A.M. Djondzo, I.N. Bass, G. Edoul, D. Meta-Djomsi, N. Lamare, C. Godwe, A. Ayouba, C. Kouanfack); University of Yaounde, Yaoundé (K. Léon); Yaoundé Central Hospital, Yaoundé (C. Tadjouteu); Cameroon National OneHealth Platform, Yaoundé (E. Dibongue); University of Dschang, Dschang, Cameroon (C. Kounanfack)

**Keywords:** Marburg virus, filovirus, Chiroptera, seroepidemiologic studies, viral haemorrhagic fever, post-outbreak surveillance, One Health, zoonoses, viruses, Cameroon, Equatorial Guinea

## Abstract

After the 2023 Equatorial Guinea Marburg virus (MARV) outbreak, surveillance of 181 persons in southern Cameroon detected MARV antibodies in 3 persons and Ebola virus antibodies in 7. Testing of 289 captured bats, including 158 *Rousettus aegyptiacus* bats, did not detect MARV RNA. Enhanced surveillance for regional filovirus spillover risks is warranted.

Marburg virus (MARV) and Ravn virus cause Marburg virus disease (MVD), a severe hemorrhagic fever with high case-fatality rates. MARV was first identified in 1967, and 20 MVD outbreaks have been documented since and are increasing in frequency (*1*). The Egyptian rousette bat (*Rousettus aegyptiacus*) is a natural reservoir for both viruses (*2*). Several outbreaks were epidemiologically linked to exposures in caves or mines harboring rousette bat colonies (*3*,*4*). The source of many index cases, including the 2023 Equatorial Guinea outbreak, remains unidentified (*5*).

On February 13, 2023, Equatorial Guinea declared its first MVD outbreak in Kie-Ntem Province with 15 confirmed cases and 11 deaths, a 73% case-fatality rate; another 23 probable cases all resulted in death. The cases occurred across 5 transmission chains from 1 viral infection. The outbreak ended on June 8, 2023 (*5*,*6*).

Southern Cameroon shares a border and extensive cross-border movements with Kie-Ntem Province, but MVD risk in Cameroon remains undefined. We conducted integrated One Health surveillance in Cameroon border communities to assess filovirus circulation risk through human seroprevalence surveys, bat sampling, and environmental investigations.

## The Study

**Figure 1:**
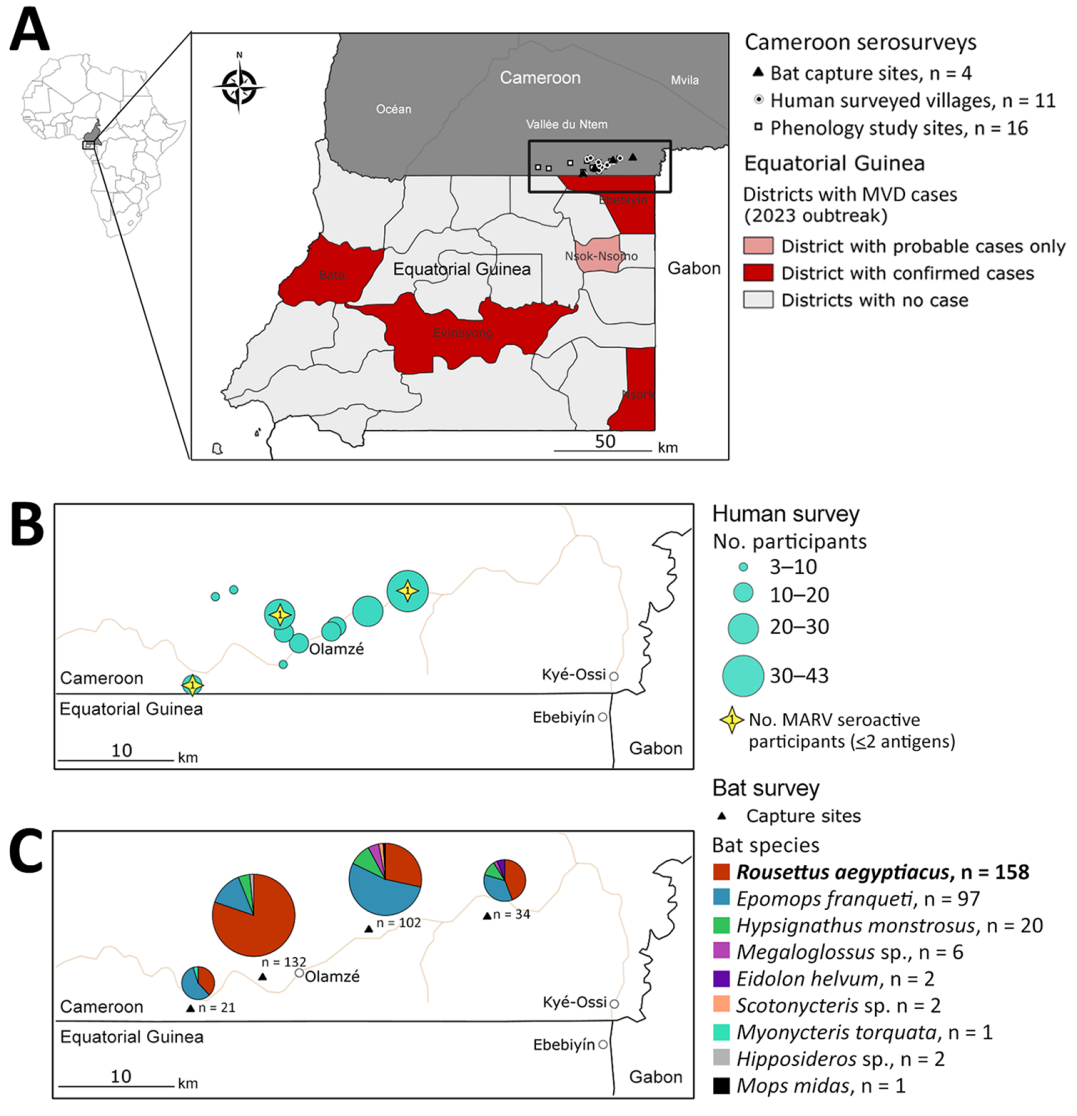
Study area of One Health surveillance for MARV in communities bordering Equatorial Guinea Marburg outbreak, Cameroon, 2023. A) Locations in Equatorial Guinea with confirmed and probable MVD cases during the outbreak (*5*) and study area in Cameroon (black box) with integrated surveillance sites (triangle, circle, and square icons). Inset map shows location of study area in Africa. B) Human survey villages; circle size indicates number of participants (n = 181 total participants). Stars indicate villages with MARV seropositive participants, defined as reactivity to >2 antigens (1.7%, n = 3 participants). C) Bat capture sites with species composition (n = 288 bats). *Rousettus aegyptiacus* bats, the MARV reservoir species, were most abundant (54.9%, n = 158 bats). We did not detect MARV RNA by using panfilovirus reverse transcription PCR of 589 bat samples. MARV, Marburg virus; MVD, Marburg virus disease.

We conducted cross-sectional seroepidemiologic surveys in 14 villages and settlements in the Olamze district, southern Cameroon, at the Equatorial Guinea border, during July–August 2023, ≈1 month after the outbreak conclusion (Figure 1; Appendix Figure 1). We enrolled 181 volunteer participants through household visits. We documented symptoms and MARV exposures that occurred 3 weeks before enrollment. We detected filovirus-specific antibodies by using a multiplex bead-based immunoassay (*7*). For MARV antigens (nucleoprotein [NP], glycoprotein 1 [GP1], and 40-kDa viral protein [VP40]), we established cutoffs as mean +3 SD of 92 seronegative reference samples from unexposed persons. We defined human seropositivity as reactivity to >2 antigens above established cutoffs (Appendix Table 1). 

Volunteer participants were a mean age of 48 years (range 6–80 years); 76 (42%) were female and 105 (58%) male. Recent symptoms were reported by 27% (n = 49) of participants; 13% (n = 24) reported fever, 12% (n = 21) reported headaches, 10% reported myalgia, and 9% reported extreme fatigue. One child reported mild epistaxis (0.6%) (Appendix Table 2).

Ten percent (n = 18) of participants traveled to Equatorial Guinea within 3 weeks before enrollment. Four (2.2%) participants had contact with persons experiencing fever, fatigue, bloody vomiting, or diarrhea; none of those persons were known or suspected MVD cases. Five (2.8%) participants provided care to sick or deceased persons, and 3 (1.7%) participants attended funerals.

Recent animal contact was reported by 45% (n = 82) of participants, most frequently with rodents (34%), pigs (18%), and nonhuman primates (12%). Common activities included collecting forest fruits (75%), butchering bushmeat (59%), and eating fruits previously bitten by animals (49%). Twenty-seven percent of participants had consumed animals found dead; 8% reported hunting animals. Bat-specific exposures included direct bat contact in the house or forest (7.7%), visiting caves (6.6%), eating bat meat (5%), and collecting guano (4%). Fruit trees surrounded 91% of participants’ homes; the African plum, avocado, papaya, and mango were the most common. Bats were observed in peridomestic trees by 62% of participants; 35% of participants collected fruits when bats were active. Botanical surveys identified 71 plant species serving as bat food sources or roosting sites (Appendix Figure 4).

Of the participants, 3 (1.7%; 95% CI 0.43%–5.2%) were MARV-seropositive: 1 was positive for NP and GP1 and 2 to GP1 and VP40. All were men, 59–79 years of age; none reported symptoms. Among the 3 MARV-seropositive participants, 1 reported funeral attendance, 2 had contact with nonhuman primates, and 1 had traveled to Equatorial Guinea. However, the small sample size precluded formal risk factor analysis (Appendix Table 2). Seropositivity to other filoviruses included 7 (3.9%, 95% CI 1.7%–8.1%) to Ebola virus (EBOV), 2 (1.1%, 95% CI 0.19%–4.4%) to Sudan virus, and 1 (0.6%, 95% CI 0.03%–3.5%) to Bundibugyo virus (Appendix Table 3). We did not find MARV cross-reactivity with EBOV or Bundibugyo virus (Appendix Figure 3).

For bat testing, we captured 289 bats over 25 nights at 4 sites by using mist nets. We performed panfilovirus reverse transcription PCR screening by using a seminested PCR targeting a 630 bp fragment of the mature light chain gene (*8*). We used a bat serology–adapted multiplex bead-based immunoassay protocol with cutoffs defined as mean +4 SD of 150 negative control samples (*9*) (Appendix). 

Among 289 captured bats, most were frugivorous species: *R. aegyptiacus* (55%, n = 158), *Epomops franqueti* (34%, n = 97), *Hypsignathus monstrosus* (7%, n = 20), and *Megaloglossus woermanni* (2%, n = 6). Other bat species included *Scotonycteris* spp. (n = 2), *Eidolon helvum* (n = 2), *Myonycteris torquata* (n = 1), *Hipposideros cyclops* (n = 2), and *Mops midas* (n = 1). *R. aegyptiacus* bats were captured at all sites and abundant in Embe-eto and Olamze (Figure 1). Caves and rock fissures in >12 investigated villages housed primarily insectivorous colonies. Sixteen of 589 samples (8/287 oral swab, 8/286 rectal swab, and 0/16 organ [8 liver, 8 spleen] samples from euthanized *R. aegyptiacus* bats) from 15 of 287 bats were positive by using panfilovirus reverse transcription PCR: 2.5% of *R. aegyptiacus* bats (n = 4/158), 9.5% of *E. franqueti* bats (n = 9/95), 5% of *H. monstrosus* bats (n = 1/20), and 50% of *E. helvum* bats (n = 1/2). However, MinION sequencing (Oxford Nanopore, https://nanoporetech.com) did not detect MARV, indicating nonspecific amplification.

**Figure 2:**
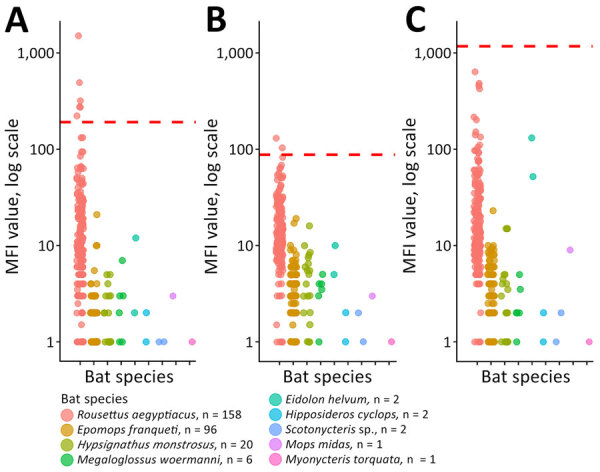
Marburg virus antibody responses in bat species in communities bordering Equatorial Guinea Marburg outbreak, Cameroon, 2023. MFI values for Marburg virus antigens nucleoprotein (A), glycoprotein 1 (B), and 40-kDa viral protein (C) are shown for 9 bat species. The red dashed horizontal line represents the seropositivity threshold, derived from log-transformed negative-control MFI values (mean +4 SD; n = 150) and back-transformed to the original MFI scale (nucleoprotein, 191; glycoprotein 1, 88; 40-kDa viral protein, 1,173). MFI, median fluorescence intensity.

MARV serologic screening of bat dried blood spots revealed that, among 158 *R. aegyptiacus* bats, 6 (3.8%, 95% CI 1.7%–8%) had antibodies against NP and 2 (1.3%, 95% CI 0.3%–4.5%) had antibodies against GP1 (Table; Figure 2). However, samples were not seropositive for both antigens simultaneously, and none were reactive to VP40. All samples from other bat species were seronegative (Appendix Table 4).

**Table:** MARV antibody responses in captured bats during filovirus surveillance in communities bordering Equatorial Guinea Marburg outbreak, Cameroon, 2023*

MARV antigens	Bat species

*Rousettus aegyptiacus*,† n = 158			Other species,‡ n = 130	
MFI, median (range)	No. (%)	MFI, median (range)	No. (%)
Nucleoprotein	11.5 (1–1,509)	6 (3.8)		2 (1–21)	0
Glycoprotein 1	13.2 (1–130)	2 (1.3)		4 (1–19)	0
40-kDa viral protein	15 (1–636)	0		3 (1–131)	0
>2 antigens	NA	0		NA	0

*No. (%) indicates samples above seropositivity thresholds. MARV, Marburg virus; MFI, Median fluorescence intensity; NA, not applicable. †Natural reservoir species for MARV. ‡Includes *Epomops franqueti* (n = 96), *Hypsignathus monstrosus* (n = 20), *Megaloglossus woermanni* (n = 6), *Eidolon helvum* (n = 2), *Hipposideros cyclops* (n = 2), *Scotonycteris* sp. (n = 2), *Mops midas* (n = 1), *Myonycteris torquata* (n = 1). No bat samples were reactive to >2 antigens, the criterion for seropositivity in human samples.

## Conclusions

We detected MARV antibodies in 3 (1.7%) men in southern Cameroon, suggesting prior exposure. One seropositive participant reported funeral attendance, a documented risk factor in the Equatorial Guinea outbreak (*5*), but small sample sizes precluded meaningful risk factor analysis, and the lack of validated positive controls limit serologic interpretation. EBOV seroprevalence (3.9%) exceeded that of MARV (1.7%), and we did not observe cross-reactivity between antigens, suggesting distinct exposures. Seroreactivity to orthoebolaviruses was previously described in rural Cameroon populations, without documented disease outbreaks (*10*). Consistent with our findings, this seroreactivity suggests ongoing filovirus exposure, highlighting regional spillover risk.

High-risk behaviors were common: half the participants consumed partially eaten fruits (*11*), and many engaged in hunting handled already dead animals. Bat meat consumption was lower than in other Cameroon regions (*12*,*13*), suggesting fruit-mediated transmission might predominate locally. However, our findings should be interpreted with caution, considering volunteer recruitment bias and missing exposure data that might limit generalizability to the broader community.

Despite the presence of *R. aegyptiacus* bats, the natural MARV reservoir (*2*), we did not detect viral RNA in the 589 bat samples we collected during the 5–11 months after the Equatorial Guinea outbreak. Negative findings could be explained by seasonal and nonpersistent viral shedding patterns (*14*) or preferential detection in organs versus swab samples (*2*,*15*). Although the reactivity to MARV antigens was higher in *R. aegyptiacus* bats compared with other bat species (Figure 2), none were reactive to >2 antigens. This low multiantigen reactivity suggests sporadic MARV circulation in *R. aegyptiacus* bats (*14*).

In summary, the presence of reservoir species, extensive cross-border movement, high-risk behaviors, and evidence of filovirus circulation in this region underscore the need for sustained One Health surveillance. Such surveillance can enable early outbreak detection and response.

Appendix Additional information about filovirus surveillance in communities bordering Equatorial Guinea Marburg outbreak, Cameroon, 2023.
