# Supplementary material for: Filovirus Surveillance in Communities Bordering Equatorial Guinea, Marburg Outbreak, Cameroon, 2023
Source: Emerg Infect Dis. 2026 Aug;32(8):1306–10. doi: 10.3201/eid3208.260117 (PMC13426847; doi:10.3201/eid3208.260117)
Supplement: Appendix — Additional information about filovirus surveillance in communities bordering Equatorial Guinea Marburg outbreak, Cameroon, 2023. [file 26-0117-Techapp-s1.pdf]

Article DOI: https://doi.org/10.3201/eid3207.260117

EID cannot ensure accessibility for supplementary materials supplied by authors. Readers who have difficulty accessing supplementary content should contact the authors for assistance.

Filovirus Surveillance in Communities Bordering Equatorial Guinea Marburg Outbreak, Cameroon, 2023

Appendix

Appendix Methods

Study Design and Setting

This post-outbreak surveillance was conducted in southern Cameroon at the border with Equatorial Guinea following the February 2023 Marburg virus disease outbreak. The MVD outbreak in Equatorial Guinea was declared on February 13, 2023, and officially ended on June 8, 2023. The first mission (July-August 2023), beginning 7 weeks after the outbreak conclusion, integrated human surveys and plant phenology assessments in Adjap-Eyinantoum (Olamze district). Four bat capture sessions were conducted in June 2023 in Adjap-Eyinantoum (Olamze), in August 2023 at Embe-eto and Meyo-Nkolayet (both Olamze district), and in December 2023 at Mekomo II (Kye-ossi district) (Appendix Figure 1).

Ethical Considerations

This study received ethical clearance from Comité National d’Ethique du Cameroun (approval N°007/CRERSH SUD/SE/2023). All participants provided written informed consent before enrolment. For minors (<21 years as per Cameroon law), written consent was obtained from parents or legal guardians, with participant assent.

Human Surveillance

Participant Recruitment and Data Collection. Active surveillance was conducted in 14 villages and settlements across three health districts (Olamze, Kye-Ossi, and Ambam) during July-August 2023. The study was conducted in a forested area with villages scattered into small settlements throughout the study zone. Recruitment was based on voluntary participation

following community sensitization activities. Inclusion was based on (*1*): residence in study villages (*2*), availability during household visits (*3*), willingness to participate and provide informed consent, and (*4*) consent for blood sampling procedures. No exclusion criteria were applied beyond inability to provide consent or refusal of blood sampling. No age restrictions were applied.

Trained field investigators administered structured questionnaires adapted from WHO outbreak investigation tools to consenting participants (Appendix Figure 5). The questionnaire collected information on: demographic characteristics (age, sex, village, occupation); clinical symptoms experienced in the previous 3 weeks before survey; epidemiologic risk factors including: travel to Equatorial Guinea; contact with hospitalized persons, or with known or suspected cases (individuals with fever, fatigue, bloody vomiting, or diarrhea); participation in funeral ceremonies or involvement in caring for or handling bodies of sick/deceased persons; and zoonotic exposure history (contact with bats or caves, contact with other wildlife (rodents, non-human primates), bushmeat hunting, handling, or consumption).

Biologic sample collection. Blood samples were collected on EDTA tubes. Whole blood was spotted onto Whatman 903 filter paper cards (dried blood spots, DBS) and air-dried for at least 4 hours. DBS were individually stored in sealed plastic bags with desiccant and transported to the Centre de Recherche sur les Maladies Émergentes et Ré-émergentes (CREMER) in Yaoundé for serologic analysis.

Bat Surveillance

Capture Methods and Sites. Bats were captured using mist nets deployed at four sites representing different ecologic interfaces: forest edges and clearing; village peripheries with fruit trees and near cave entrances. Nets were set before dusk and monitored throughout the night for three consecutive nights per site. Captured bats were carefully removed from nets every 30–60 minutes.

Bat Handling and Data Collection. For each captured bat, the following data were recorded: species identification using morphological keys; body measurements (forearm length, body mass); age class (adult versus juvenile); sex and reproductive status. Species were identified on the field and laboratory confirmation was made on a subset of animals. For each captured bat, oral, rectal swabs and blood on DBS were collected. After sampling, captured bats

were released at the capture site. Organs were collected from a subset of euthanized *Rousettus aegyptiacus* (n = 8) following euthanasia protocols (isoflurane overdose).

Molecular Screening for Filoviruses in Bats

Nucleic Acid Extraction. RNA was extracted from oral and rectal swabs stored in RNAlater using the QIAamp Viral RNA Mini Kit (Qiagen, Les Ulis, France). For each sample, 250 μL of RNAlater-sample mixture was processed according to the manufacturer's protocol, with final elution in 60 μL of elution buffer. Tissue samples (30 mg spleen or liver) were homogenized in 200 μL lysis buffer and 50 μL Proteinase K using a Minilys homogenizer (Bertin Technologies, France), then extracted using the GeneJET Viral DNA & RNA Purification Kit (Thermo Fisher Scientific, USA) per manufacturer's instructions. Field-based bat species identifications were molecularly confirmed in a subset of samples by sequencing an 800 bp fragment of the mitochondrial cytochrome b gene, as previously described (*1*,*2*).

Pan-Filovirus RT-PCR Screening. Complementary DNA (cDNA) was synthesized from 5 μL of extracted RNA using the Reverse Transcription System A3500 (Promega, Madison, WI, USA) with random primers according to the manufacturer's protocol. Pan-filovirus screening was performed using a broadly reactive semi-nested RT-PCR targeting a 630 bp fragment of the RNA-dependent RNA polymerase (L) gene, as previously described (*1*,*3*). The assay employs degenerate primers designed to detect a wide range of filoviruses including Marburg, Ebola, and related viruses.

PCR amplification was performed using GoTaq Hot Start Master Mix (Promega, Madison, WI, USA) under the following cycling conditions:

- First round PCR: Initial denaturation at 95°C for 2 min; 10 cycles of 92°C for 20 s, 50°C for 30 s (with –0.5°C per cycle), and 72°C for 1 min; followed by 35 cycles of 92°C for 20 s, 50°C for 30 s, and 72°C for 1 min; final extension at 72°C for 5 min.

- Second round PCR (semi-nested): Same cycling conditions using 1 μL of first-round product as template with nested primers. PCR products were analyzed by 1.5% agarose gel electrophoresis.

Nanopore Sequencing. Amplicons of expected size (~630 bp) were prepared for ligation sequencing using the Native Barcoding Kit 24 V14 (EXP-NBD114.24, Oxford Nanopore

Technologies, Oxford, UK). Libraries were loaded onto a R10 FLO-MIN112 flow cell and sequenced on a MinION Mk1C device (Oxford Nanopore Technologies, Oxford, UK) for 24–48 hours. Base-calling, adaptor removal, and demultiplexing were performed in real-time using MinKNOW software (version 23.04.5). Consensus sequences were generated using Medaka (version 1.7.2) and taxonomically assigned using BLAST against the NCBI nucleotide database.

Serologic Detection of Filovirus Antibodies

Multiplex Bead-Based Immunoassay (MIA). Filovirus-specific antibodies were detected in human and bat dried blood spots (DBS) using a multiplex bead-based immunoassay, on a Luminex plateform as previously described for human (*4*) and bat (*5*) samples. Whole blood was reconstituted from DBS punches and inactivated as previously described (*6*). The assay included 14 recombinant filovirus antigens: nucleoprotein (NP), glycoprotein (GP), and viral protein 40 (VP40) for Marburg virus (MARV) and four orthoebolavirus species: Ebola virus (EBOV), Sudan virus (SUDV), Bundibugyo virus (BDBV), and Reston virus (RESTV). MARV antigens included NP and VP40 (Cusabio Technology, Houston, TX, USA) and GP1 (Native Antigen Company, Oxford, UK).

Reconstituted samples were diluted to a final plasma dilution of 1:1000 (human) or 1:2000 (bat) and 100 µL of diluted sample was incubated with 50 µL of antigen-coupled magnetic beads in 96-well flat-bottom chimney plates (Greiner bio one, Frickenhausen, Germany) for 16 hours at 4°C on a plate shaker at 300 rpm in the dark. After washing, bound antibodies were detected using biotin-conjugated anti-human IgG (Jackson ImmunoResearch, West Grove, PA, USA) for human samples or goat anti-bat biotin–labeled IgG (Euromedex, Souffelweyersheim, France) for bat samples. Following a second wash, 50 µL of streptavidin-R-phycoerythrin (4 µg/mL; Fisher Scientific/Life Technologies, Illkirch, France) was added per well and incubated for 10 min at 300 rpm at room temperature. Median fluorescence intensity (MFI) was measured using a BioPlex-200 (BioRad, Marnes-la-Coquette, France) or MagPix (Luminex, Austin, TX, USA) system, with a minimum of 50 beads counted per antigen.

Seropositivity Cutoff Determination

Orthoebolaviruses (human samples): cutoffs were previously determined by ROC curve analysis, using validated EBOV-positive samples (plasma from 2014–2016 Guinea EBOV outbreak survivors, n = 94) and EBOV-negative samples (sera from French unexposed patients,

n = 108) (*4*,*7*). These EBOV-derived cutoffs were applied to all orthoebolavirus species (NP = 600; GP = 450; VP40 = 650). A sample was considered seropositive if MFI exceeded the cutoff value for at least two antigens (NP, GP, or VP40) of a given virus species.

MARV (human samples): In the absence of confirmed MVD survivor sera for ROC analysis, seropositivity thresholds for MARV antigens were defined as the mean + 3 standard deviations (SD) of MFI values from 92 seronegative reference samples collected from healthy individuals in Yaoundé, Cameroon, with no known filovirus exposure or travel to outbreak-affected regions. Cutoffs were: NP = 1,448; GP1 = 331; VP40 = 1,141. Seropositivity was defined as MFI above threshold for at least two antigens (any combination of NP, GP1, and VP40).

MARV (bat samples): Cutoffs for MARV antigens for bat samples were defined as the mean + 4 SD of MFI values from 150 negative control samples from 105 captive-born insectivorous bats (103 *Carollia perspicillata* bats) hosted at the Parc Zoologique de Montpellier (Montpellier, France) and from 45 frugivorous bats (18 *Pteropus giganteus* bats, 27 *R. aegyptiacus* bats) hosted at Wilhelma Zoo and Botanical Garden (Stuttgart, Germany), as previously used (*5*). Because raw MFI values were right-skewed, antigen-specific cutoffs were defined on log-transformed MFI values as the mean plus four standard deviations and then back-transformed to the original MFI scale. MFI values differed by bat family, with frugivorous *Rousettus aegyptiacus* showing significantly higher baseline reactivity for GP1 and VP40 antigens compared to other bat species from the negative pool. We conducted sensitivity analyses using species-specific seropositivity cutoffs. Bootstrap confidence intervals for species-specific cutoffs exceeded 85% relative width and overlapped between species, particularly for *R. aegyptiacus* (n = 27) precluding reliable species-specific cutoff determination. Therefore, we used pooled cutoffs derived from all negative controls (n = 150) for analyses.

Statistical Analysis

Descriptive statistics were calculated for demographic and exposure variables. Seroprevalence rates with 95% confidence intervals (95%CI) were calculated for overall seropositivity and stratified by characteristics. Proportions were compared using Fisher exact test. Statistical significance was set at $\alpha = 0.05$. Logistic regression and odds ratio calculations

were not performed due to the small number of seropositive cases (n = 3). All analyses were performed using R version 4.3.1 (R Core Team, 2023).

Plant Phenology Survey

Site Selection and Sampling Design. Preliminary consultations were conducted with community members in villages of the Olamze health district to gather local knowledge on: bat presence and roosting sites in or near villages; fruit tree species known or observed to be consumed by bats; seasonal patterns of fruiting and bat visitation. These consultations informed the selection of survey sites and targeted plant species for the floristic inventory. Two complementary methods were used for plant surveys:

1. Plot-based inventory: Rectangular plots measuring 25 m × 20 m (500 m^2) were established along existing paths and trails within and around villages. A total of 51 plots covering 2.68 ha were surveyed. Plots were stratified across six habitat types: home gardens, cocoa plantations, secondary forests, swamp forests, riparian forests, and seasonally flooded forests.

2. Walk-through surveys: Appendix itinerant surveys were conducted along transects to record additional species not captured in fixed plots, particularly roosting trees and fruiting trees reported by community members.

Data Collection. Within each plot, all woody plants (trees and large shrubs) were recorded and identified to species level by trained botanists with assistance from local guides. For each individual plant, the following information was documented: Scientific and local names, abundance (number of individuals per plot), habitat type and functional category: Food plant (Fruit-bearing species known or observed to be consumed by frugivorous bats) or roosting sites (Trees used by bats as diurnal roosts, based on direct observation, bat feces, local knowledge and literature) (*8–10*).

Data Analysis. Species richness (number of species per village), abundance (number of individuals per species), and habitat preferences were calculated. Villages were categorized by ecologic function (feeding sites versus roosting sites) based on the relative abundance of food plants versus roosting trees. This information was used to identify high-risk villages where human-bat contact interfaces are most likely to occur.

MARCAM project participants

NDONG BASS Innocent ; Centre de Recherches sur les Maladies Emergentes, Re-émergentes, Yaoundé, Cameroon ; FOTSOP Thomas CREMER ; MOUDINDO Joseph CREMER ; WOGUIA Gilles-Fils CREMER; Docteur KONO Léon Dieudonné, Enseignant de Botanique-Ecologie à l'Université de Yaoundé I ; Dr FOUOGUE Wilfred, Programme National de Prévention et de Lutte contre les Zoonoses Emergentes et Réémergentes ; Dr MAYONE ZINTCHEM Roussel, cadre Délégation Régional du MINFOF-SUD ; Dr METCHOP Armelle, Chef de District de Santé AMBAM ; M MOUANGUE Christian, Data Manager du Centre de Coordination des Urgences de Santé Publique (CCOUSP) ; M NGU FANKAM Roland, Chef District de Santé d'Olamzé ; Dr EBANGA Franck, Chef de District de Santé Kye-Ossi ; MAMA ESSO Gaëtan Marquise, cadre au District de santé d'Olamzé ; ESSISSIMA ESSONO Bertrand, Agent de Santé Communautaire ; Olamzé ; NDONG ONDO Engelbert, Point focal communication au District de Santé d'Olamzé ; Dr NGONGLA NGONGLA Firmin Néhémie, Coordonnateur Centre Régional de Prévention et de Lutte contre les Zoonoses, Délégation Régionale MINSANTE-SUD ; TEME Joseph Magloire, étudiant Université de Dschang ; NDEFO FOKAM Alex Brise, étudiant Université de Dschang ; NUMVI Collins, Programme National de Prévention et de Lutte contre les Zoonoses Emergentes et Réémergentes ; ADOUEME OLAGA Manassé Hermann, secrétaire permanent du Réseau des Organisations One Health du Cameroun (ROOHCAM) ; AYEBONJE Rolland, Direction des Services Vétérinaires, Ministère de l'Elevage, des pêches et industries animales (MINEPIA) ; ZANG ZANG Martin Alain, technicien principal des eaux et forêts, Délégation Régionale du Ministère de l'Environnement, de la Protection de la Nature et du Développement Durable (MINEPDED), Région du SUD ; Dr ELOUNDOU NKA Marc-Cyrille, Point focal One Health, Organisation Mondiale de la Santé.

References

1. Lacroix A, Mbala Kingebeni P, Ndimbo Kumugo SP, Lempu G, Butel C, Serrano L, et al. Investigating the circulation of Ebola viruses in bats during the Ebola virus disease outbreaks in the Equateur and North Kivu Provinces of the Democratic Republic of Congo from 2018. Pathogens. 2021;10:557. PubMed https://doi.org/10.3390/pathogens10050557

2. Irwin DM, Kocher TD, Wilson AC. Evolution of the cytochrome b gene of mammals. J Mol Evol. 1991;32:128–44. PubMed https://doi.org/10.1007/BF02515385

3. Goldstein T, Anthony SJ, Gbakima A, Bird BH, Bangura J, Tremeau-Bravard A, et al. Discovery of a new ebolavirus (Bombali virus) in molossid bats in Sierra Leone. Nat Microbiol. 2018;3:1084–9. PubMed https://doi.org/10.1038/s41564-018-0227-2

4. Ayouba A, Touré A, Butel C, Keita AK, Binetruy F, Sow MS, et al. Development of a sensitive and specific serological assay based on luminex technology for detection of antibodies to Zaire Ebola virus. J Clin Microbiol. 2016;55:165–76. PubMed https://doi.org/10.1128/JCM.01979-16

5. De Nys HM, Kingebeni PM, Keita AK, Butel C, Thaurignac G, Villabona-Arenas CJ, et al. Survey of Ebola viruses in frugivorous and insectivorous bats in Guinea, Cameroon, and the Democratic Republic of the Congo, 2015–2017. Emerg Infect Dis. 2018;24:2228–40. PubMed https://doi.org/10.3201/eid2412.180740

6. Meta Djomsi D, Lacroix A, Soumah AK, Kinganda Lusamaki E, Mesdour A, Raulino R, et al. Coronaviruses are abundant and genetically diverse in West and Central African bats, including viruses closely related to human coronaviruses. Viruses. 2023;15:337. PubMed https://doi.org/10.3390/v15020337

7. Keita AK, Vidal N, Toure A, Diallo MSK, Magassouba N, Baize S, et al.; PostEbogui Study Group. A 40-month follow-up of ebola virus disease survivors in guinea (postebogui) reveals long-term detection of Ebola viral ribonucleic acid in semen and breast milk. Open Forum Infect Dis. 2019;6:ofz482. PubMed https://doi.org/10.1093/ofid/ofz482

8. Rakotondramanana CF, Rajemison B, Goodman SM. Feeding behaviour of the animalivorous bat communities of Kirindy (CNFEREF) and Antsahabe, Madagascar: distribution, sharing and availability of food niches [in French]. Malagasy Nature. 2015;9:68–87.

9. Nurul-Ain E, Rosli H, Kingston T. Resource availability and roosting ecology shape reproductive phenology of rain forest insectivorous bats. Biotropica. 2017;49:382–94. https://doi.org/10.1111/btp.12430

10. Niamien CJM, Kadjo B, Kone I. Preliminary data on the ecology of fruit bats in the commune of Plateau (Abidjan, Côte d'Ivoire) [in French]. Afrique science. 2015;11(1):227–236

Appendix Table 1. Filovirus-specific antibody reactivity in human participants

Filovirus	Antigen	Median (Min-Max)	n (%)
MARV	NP	96 (10–6,314)	4 (2.2%)
	GP1	15 (2–3,269)	12 (6.6%)
	VP40	156 (40–3,520)	8 (4.4%)
EBOV	NP	56 (10–742)	3 (1.7%)
	GP.kiss	212 (11–1,811)	31 (17%)
	GP.may	75 (4–3,286)	26 (14%)
	VP40	132 (20–11,896)	29 (16%)
SUDV	NP	40 (4–2,387)	4 (2.2%)
	GP1	119 (7–3,468)	30 (17%)
	VP40	94 (10–1,886)	5 (2.8%)
BDBV	GP	45 (3–878)	7 (3.9%)
	VP40	76 (14–8,993)	12 (6.6%)
RESTV	GP	21 (1–565)	1 (0.6%)
BOMV	GP1	17 (0–266)	0 (0%)

Values represent median (minimum-maximum) of median fluorescence intensity (MFI) among 181 participants from Cameroon. n (%): number of reactive antigens (percentage). Reactivity is defined as MFI above the following thresholds: MARV NP = 1,448; MARV GP1 = 331; MARV VP40 = 1,141; *orthoebolaviruses* NP = 600; *orthoebolaviruses* GP = 450; *orthoebolaviruses* VP40 = 650.

Appendix Table 2. Demographics and exposure history among study participants by MARV serologic results

	Overall N(%)	MARV Reactive Serology†		
Characteristic	Total = 181	n/N	% (95% CI)	p-value‡
DEMOGRAPHICS				
Sex				0.3
F	76 (42%)	0/76	0% (0–6.0)	
M	105 (58%)	3/105	2.9% (0.7–8.7)	
Age category				0.2
<35	51 (28%)	0/51	0% (0–8.7)	
35–50	44 (24%)	0/44	0% (0–10)	
50–65	44 (24%)	2/44	4.5% (0.8–16.7)	
>65	42 (23%)	1/42	2.4% (0.1–14.1)	
CLINICAL SYMPTOMS*				
Any symptoms				0.6
No	94 (52%)	2/94	2.1% (0.4–8.2)	
Yes	49 (27%)	0/49	0% (0–9.1)	
NA	38 (21%)	1/38	2.6% (0.14–15.4)	
Fever				>0.9
No	131 (72%)	3/131	2.3% (0.59–7.06)	
Yes	24 (13%)	0/24	0% (0–17.2)	
NA	26 (14%)	0/26	0% (0–16.0)	
Headache				>0.9
No	118 (65%)	2/118	1.7% (0.3–6.6)	
Yes	21 (12%)	0/21	0% (0–19.2)	
NA	42 (23%)	1/42	2.4% (0.1–14.1)	
Myalgia				>0.9
No	119 (66%)	2/119	1.7% (0.3–6.5)	
Yes	18 (9.9%)	0/18	0% (0–21.9)	
NA	44 (24%)	1/44	2.3% (0.1–13.5)	
Vomiting				0.6
No	136 (75%)	2/136	1.5% (0.3–5.7)	
Yes	2 (1.1%)	0/2	0% (0–80.2)	
NA	43 (24%)	1/43	2.3% (0.1–13.8)	
Diarrhea				0.6
No	135 (75%)	2/135	1.5% (0.3–5.8)	
Yes	4 (2.2%)	0/4	0% (0–60.4)	
NA	42 (23%)	1/42	2.4% (0.1–14.1)	
Dyspnea				>0.9
No	131 (72%)	2/131	1.5% (0.3–5.9)	
Yes	6 (3.3%)	0/6	0% (0–48.3)	
NA	44 (24%)	1/44	2.3% (0.1–13.5)	
Bleeding				>0.9
No	132 (73%)	2/132	1.5% (0.3–5.9)	
Yes	1 (0.6%)	0/1	0% (0–94.5)	
NA	48 (27%)	1/48	2.1% (0.1–12.5)	
TRAVEL AND FUNERAL ATTENDANCE*				
Travel to Equatorial Guinea				0.4
No	154 (85%)	2/154	1.3% (0.2–5.1)	

Characteristic	Overall N(%) Total = 181	MARV Reactive Serology† n/N	% (95% CI)	p-value‡
Yes	19 (10%)	1/19	5.3% (0.3–28.1)	
NA	8 (4.4%)	0/8	0% (0–40.2)	
Funeral attendance				0.007
NA	65 (36%)	2/65	3.1% (0.5–11.6)	
No	113 (62%)	0/113	0% (0–4.1)	
Yes	3 (1.7%)	1/3	33% (1.8–87.5)	
Contact with a sick individual				>0.9
No	134 (74%)	3/134	2.2% (0.6–6.9)	
Yes	4 (2.2%)	0/4	0% (0–60.4)	
NA	43 (24%)	0/43	0% (0–10.2)	
BAT EXPOSURE				
Direct contact with bats				0.3
No	159 (88%)	2/159	1.3% (0.2–4.9)	
Yes	14 (7.7%)	1/14	7.1% (0.4–35.8)	
NA	8 (4.4%)	0/8	0% (0–40.2)	
Consumed bat meat				0.2
No	159 (88%)	2/159	1.3% (0.2–4.9)	
Yes	9 (5.0%)	1/9	11% (0.6–49.3)	
NA	13 (7.2%)	0/13	0% (0–28.3)	
Collected bat guano				>0.9
No	146 (81%)	3/146	2.1% (0.5–6.4)	
Yes	8 (4.4%)	0/8	0% (0–40.2)	
NA	27 (15%)	0/27	0% (0–15.5)	
Observed bats roosting near home				0.6
No	58 (32%)	0/58	0% (0–7.7)	
Yes	112 (62%)	3/112	2.7% (0.7–8.2)	
NA	11 (6.1%)	0/11	0% (0–32.1)	
Consumed fruit partially eaten by bats				>0.9
No	68 (38%)	1/68	1.5% (0.1–9.0)	
Yes	89 (49%)	2/89	2.2% (0.4–8.6)	
NA	24 (13%)	0/24	0% (0–17.2)	
Collected palm wine				0.7
No	91 (50%)	1/91	1.1% (0.06–6.8)	
Yes	62 (34%)	2/62	3.2% (0.6–12.2)	
NA	28 (15%)	0/28	0% (0–15.0)	
Harvested wild fruit				>0.9
No	24 (13%)	0/24	0% (0–17.2)	
Yes	136 (75%)	3/136	2.2% (0.6–6.8)	
NA	21 (12%)	0/21	0% (0–19.2)	
Visited cave				0.3
No	132 (73%)	2/132	1.5% (0.3–5.9)	
Yes	12 (6.6%)	1/12	8.3% (0.4–40.2)	
NA	37 (20%)	0/37	0% (0–11.7)	
OTHER ANIMAL CONTACT				
Contact with non-human primates				0.072
No	151 (83%)	1/151	0.7% (0.03–4.2)	
Yes	22 (12%)	2/22	9.1% (1.6–30.6)	
NA	8 (4.4%)	0/8	0% (0–40.2)	
Contact with rodents				>0.9
No	111 (61%)	2/111	1.8% (0.3–7.0)	
Yes	62 (34%)	1/62	1.6% (0.08–9.8)	
NA	8 (4.4%)	0/8	0% (0–40.2)	
HUNTING AND BUSHMEAT PRACTICES				
Hunting				0.3
No	140 (77%)	2/140	1.4% (0.2–5.6)	
Yes	16 (8.8%)	1/16	6.3% (0.3–32.3)	
NA	25 (14%)	0/25	0% (0–16.6)	
Butchering				>0.9
No	56 (31%)	1/56	1.8% (0.09–10.8)	
Yes	106 (59%)	2/106	1.9% (0.3–7.3)	
NA	19 (10%)	0/19	0% (0–20.9)	
Selling bushmeat				0.7
No	100 (55%)	1/100	1.0% (0.05–6.2)	
Yes	62 (34%)	2/62	3.2% (0.6–12.2)	
NA	19 (10%)	0/19	0% (0–20.9)	
Animal carcasses found				0.5
No	147 (81%)	2/147	1.4% (0.2–5.3)	
Yes	10 (5.5%)	0/10	0% (0–34.5)	

Characteristic	Overall N(%) Total = 181	MARV Reactive Serology† n/N	% (95% CI)	p-value‡
NA	24 (13%)	1/24	4.2% (0.2–23.1)	
Ate animal carcasses found				0.7
No	110 (61%)	3/110	2.7% (0.7–8.4)	
Yes	49 (27%)	0/49	0% (0–9.1)	
NA	22 (12%)	0/22	0% (0–18.5)	
Bitten by a wild animal				0.2
No	146 (81%)	2/146	1.4% (0.2–5.4)	
Yes	9 (5.0%)	1/9	11% (0.6–49.3)	
NA	26 (14%)	0/26	0% (0–16.0)	

Participant characteristics among 181 individuals. *Self-reported data from 3 weeks before survey. †MARV-reactive serology: antibodies to ≥2 antigens. MFI values for filovirus-specific antibodies are provided in Appendix Table S1 and Figure S2. N = total participants; n = number MARV-positive; 95% CI = 95% confidence interval; NA: not available. ‡Fischer's exact test. Descriptive comparisons are presented for exploratory purposes only. Formal statistical analysis of risk factors was not performed given the small number of MARV-positive cases (n = 3).

Appendix Table 3. Filovirus seroprevalence in human participants by antigen combination

Filovirus/Antigen combination	n (seroprevalence, 95%CI)
MARV	
NP + GP + VP40	0 (0%, 0.00%–2.6%)
At least two antigens	3 (1.7%, 0.43%–5.2%)
EBOV	
NP + GP + VP40	1 (0.6%, 0.03%–3.5%)
At least two antigens	7 (3.9%, 1.7%–8.1%)
SUDV	
NP + GP + VP40	0 (0%, 0.00%–2.6%)
At least two antigens	2 (1.1%, 0.19%–4.4%)
BDBV	
At least two antigens	1 (0.6%, 0.03%–3.5%)

Number and seroprevalence (with Wilson score 95% confidence intervals) of participants from Cameroon (N = 181) showing reactivity to at least two filovirus antigens.

Appendix Table 4. MARV-specific antibody reactivity in captured bats

		NP MARV		GP1 MARV		VP40 MARV	
Bat species	n	Median MFI (range)	n+ (%)	Median MFI (range)	n+ (%)	Median MFI (range)	n+ (%)
Rousettus aegyptiacus	158	11.5 (1–1509)	6 (3.8)	13.2 (1–129.5)	2 (1.3)	15 (1–635.5)	0 (0)
Epomops franqueti	96	2 (1–21)	0 (0)	4 (1–19)	0 (0)	3 (1–23)	0 (0)
Hypsignathus monstrosus	20	1 (1–5)	0 (0)	4.5 (1–16)	0 (0)	2.5 (1–15)	0 (0)
Megaloglossus woermanni	6	2.5 (1–7)	0 (0)	3.8 (1–5)	0 (0)	2 (1–5)	0 (0)
Eidolon helvum	2	7 (2–12)	0 (0)	7.5 (5–10)	0 (0)	91.5 (52–131)	0 (0)
Hipposideros cyclops	2	1.5 (1–2)	0 (0)	1.5 (1–2)	0 (0)	1.5 (1–2)	0 (0)
Scotonycteris sp.	2	1 (1–1)	0 (0)	1.5 (1–2)	0 (0)	1.5 (1–2)	0 (0)
Mops midas	1	3	0 (0)	3	0 (0)	9	0 (0)
Myonycteris torquata	1	1	0 (0)	1	0 (0)	1	0 (0)

Median fluorescence intensity (MFI) values and seroprevalence of MARV-specific antibodies in captured bats (N = 288).

Appendix Table 5. Abundance of plant species recorded in the 16 study villages in Cameroon.

Bat use	Plant species	Family	Total abundance	Relative density (%)
Food plants				
	Musanga cecropioides	Urticaceae	31	9.37
	Uapaca guineensis	Euphorbiaceae	26	7.86
	Persea americana	Lauraceae	19	5.74
	Dacryodes edulis	Burseraceae	18	5.44
	Psidium guajava	Myrtaceae	18	5.44
	Mangifera indica	Anacardiaceae	15	4.53
	Ficus mucuso	Moraceae	10	3.02
	Myrianthus arboreus	Urticaceae	10	3.02
	Irvingia gabonensis	Irvingiaceae	9	2.72
	Spondias mombin	Anacardiaceae	7	2.11
	Musa spp.	Musaceae	6	1.81
	Annona muricata	Annonaceae	5	1.51
	Carica papaya	Caricaceae	5	1.51
	Trichocypha abut	Anacardiaceae	5	1.51
	Ficus exasperata	Moraceae	4	1.21
	Barteria fistulosa	Passifloraceae	3	0.91
	Anonidium mannii	Annonaceae	2	0.6
	Carpolobia alba	Polygalaceae	2	0.6
	Dacryodes macrophylla	Burseraceae	2	0.6
	Mammea africana	Clusiaceae	2	0.6
	Musa sapientum	Musaceae	2	0.6
	Baillonella toxisperma	Sapotaceae	1	0.3
	Ficus sp.	Moraceae	1	0.3
	Milicia excelsa	Moraceae	1	0.3
	Musa paradisiaca	Musaceae	1	0.3
	Oncoba welwitschii	Salicaceae	1	0.3
	Ongokea gore	Olacaceae	1	0.3
Roosting sites				
	Elaeis guineensis	Arecaceae	12	3.63
	Distemonanthus bentamianus	Fabaceae	9	2.72
	Petersianthus macrocarpus	Lecythidaceae	7	2.11
	Theobroma cacao	Malvaceae	7	2.11
	Eucalyptus camaldulensis	Myrtaceae	6	1.81
	Entandrophragma cylindricum	Meliaceae	5	1.51
	Cocos nucifera	Arecaceae	4	1.21
	Gilbertiodendron dewevrei	Fabaceae	4	1.21
	Pentaclethra macrophylla	Fabaceae	4	1.21
	Pycnanthus angolensis	Myristicaceae	4	1.21
	Albizia zygia	Fabaceae	3	0.91
	Cola filicifolia	Malvaceae	3	0.91
	Fagara sp.	Rutaceae	3	0.91
	Hallea stipulosa	Rubiaceae	3	0.91
	Macaranga assas	Euphorbiaceae	3	0.91
	Pterocarpus soyauxii	Fabaceae	3	0.91
	Raphia mambillensis	Arecaceae	3	0.91
	Ricinodendron heudelotii	Euphorbiaceae	3	0.91
	Tetrapleura tetraptera	Fabaceae	3	0.91
	Alstonia boonei	Apocynaceae	2	0.6
	Ceiba pentandra	Malvaceae	2	0.6
	Piptadeniastrum africanum	Fabaceae	2	0.6
	Sterculia tragacantha	Malvaceae	2	0.6
	Strombosia pustulata	Olacaceae	2	0.6
	Tabernaemontana crassa	Apocynaceae	2	0.6
	Trema orientalis	Ulmaceae	2	0.6
	Triplochiton scleroxylon	Malvaceae	2	0.6
	Albizia ferruginea	Fabaceae	1	0.3
	Amphimas pterocarpoides	Fabaceae	1	0.3
	Anthocleista schweinfurthii	Loganiaceae	1	0.3
	Anthonotha fragrans	Fabaceae	1	0.3
	Bridelia micrantha	Euphorbiaceae	1	0.3
	Celtis zenkeri	Ulmaceae	1	0.3
	Cylicodiscus gabonensis	Fabaceae	1	0.3
	Enantia chloranta	Annonaceae	1	0.3
	Erythroxylum mannii	Erythroxylaceae	1	0.3
	Guarea cedrata	Meliaceae	1	0.3
	Lophira alata	Ochnaceae	1	0.3
	Psydrax sp.	Rubiaceae	1	0.3

Bat use	Plant species	Family	Total abundance	Relative density (%)
	Santiria trimera	Burseraceae	1	0.3
	Spathodea campanulata	Bignoniaceae	1	0.3
	Terminalia superba	Combretaceae	1	0.3
	Vepris natalensis	Rutaceae	1	0.3
	Vepris sp.	Rutaceae	1	0.3

Abundance of plant species recorded across 16 villages in Olamzé district (N = 329 individual trees). Relative density = (number of stems/ha of species i) / (total stems/ha) × 100. Functional category indicates whether species serve as food plants (fruit-bearing species consumed by frugivorous bats) or roosting sites (trees used by bats for shelter).

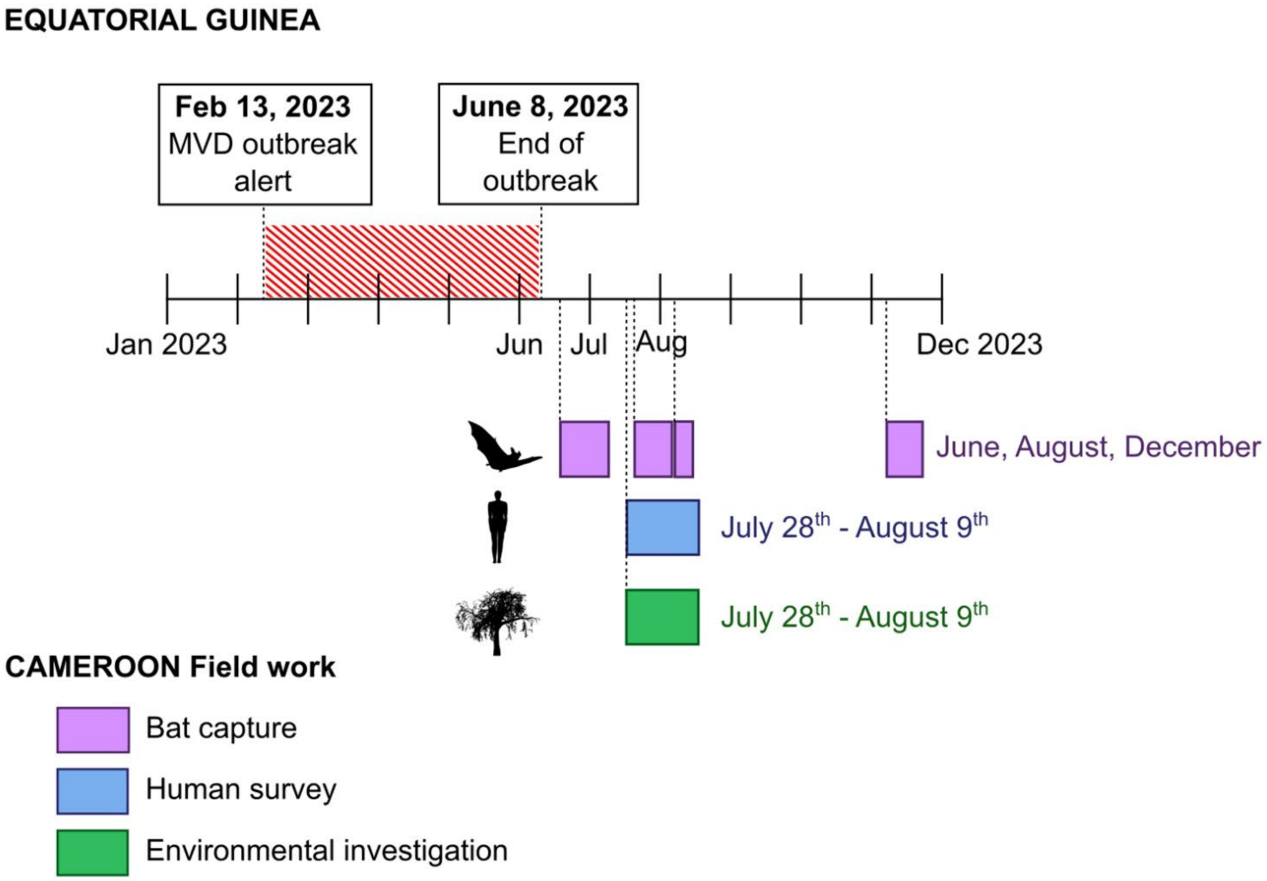

Appendix Figure 1. Timeline of Equatorial Guinea Marburg virus disease outbreak and Cameroon surveillance activities, 2023. Timeline showing the Equatorial Guinea MVD outbreak (February 13 - June 8, 2023, red hatched area) and Cameroon surveillance activities, including bat sampling (purple), human serosurveys (blue), and environmental investigations (green).

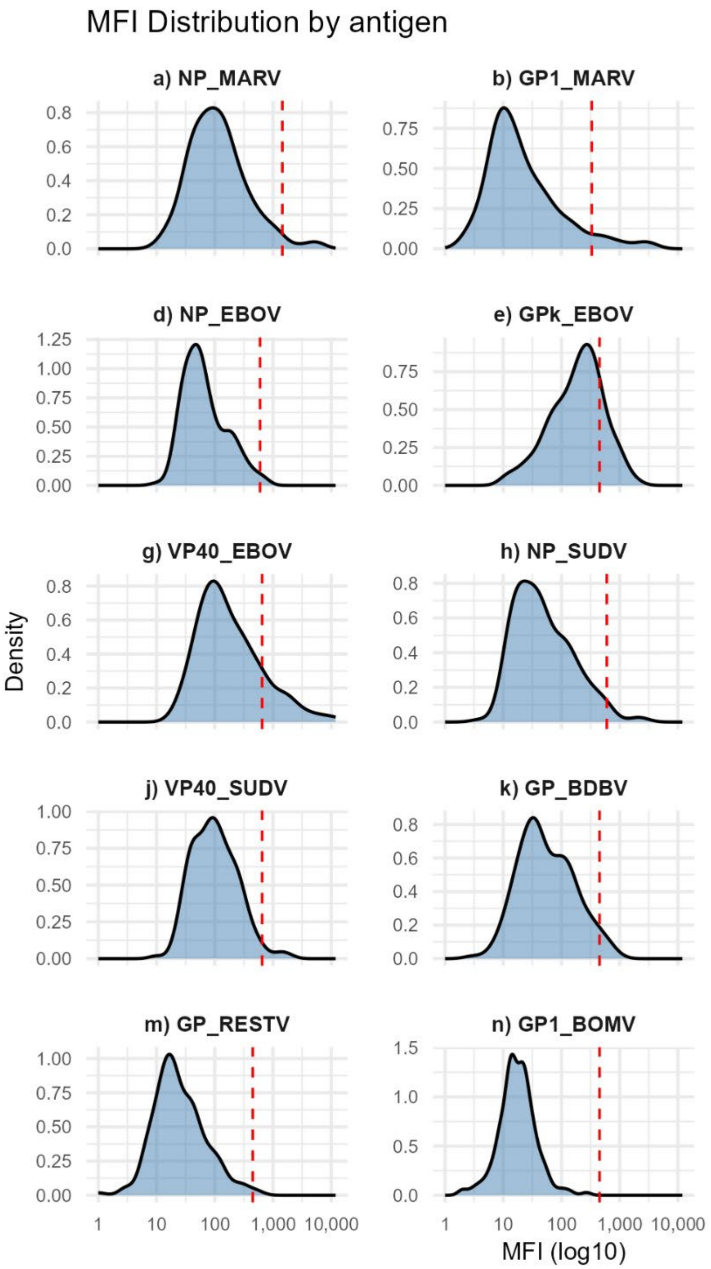

Appendix Figure 2. MFI distributions for filovirus antigens. Density plots showing Median Fluorescence Intensity (MFI) distributions for filovirus antigens tested in study participants (n = 181). Red dashed lines indicate seropositivity cutoffs. Cutoffs were determined determined by mean + 3SD of negative controls for MARV antigens (NP = 1,448; GP1 = 331; VP40 = 1,141) and by ROC curve analysis for EBOV antigens (NP = 600; GP = 450; VP40 = 650); EBOV-derived cutoffs were applied to all *orthoebolavirus* species. Abbreviations: BDBV, Bundibugyo virus; BOMV, Bombali virus; EBOV, Ebola virus; GP, glycoprotein; GP1, glycoprotein 1; MARV, Marburg virus; MFI, median fluorescence intensity; NP, nucleoprotein; RESTV, Reston virus; SUDV, Sudan virus; VP40, viral protein 40.

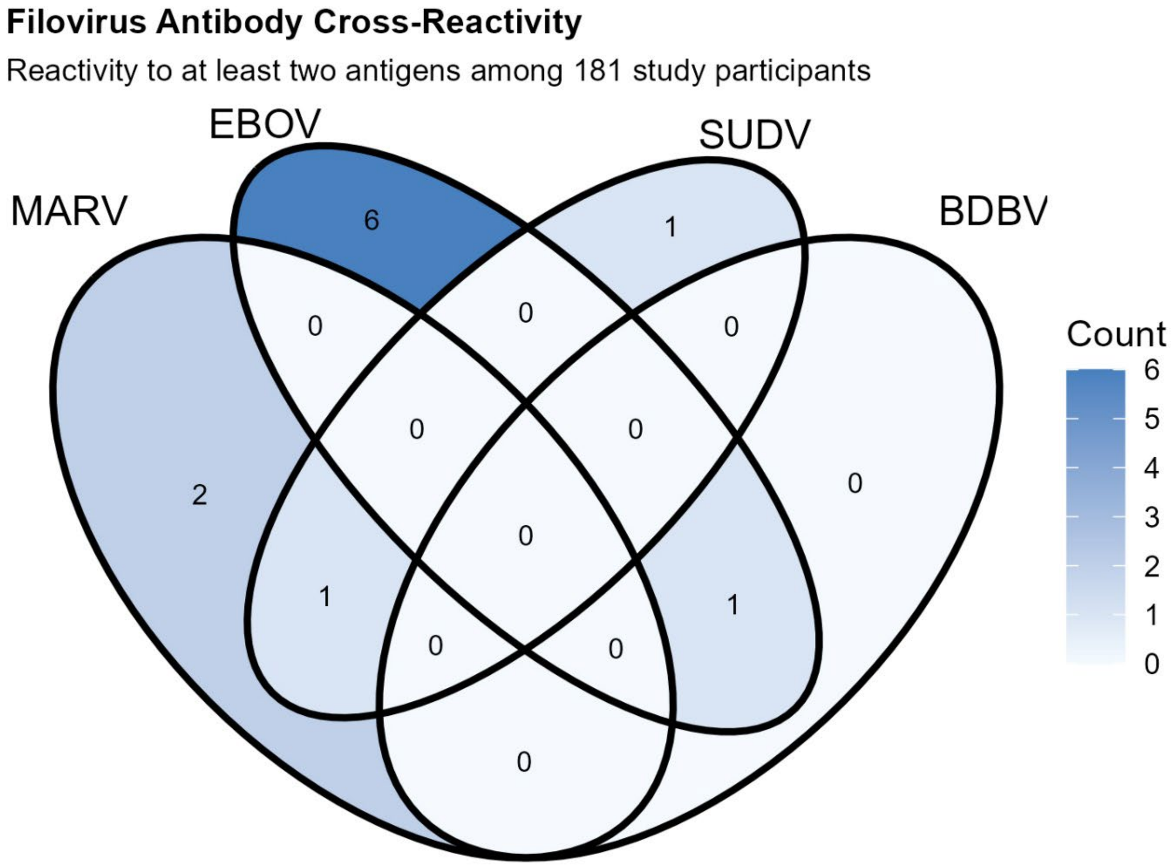

Appendix Figure 3. Filovirus cross-reactivity. Venn diagram showing overlap of seropositive individuals (N = 181) for MARV, EBOV, SUDV, and BDBV. Seropositivity defined as reactivity to at least two antigens (NP, GP, and/or VP40). Numbers indicate participant counts.

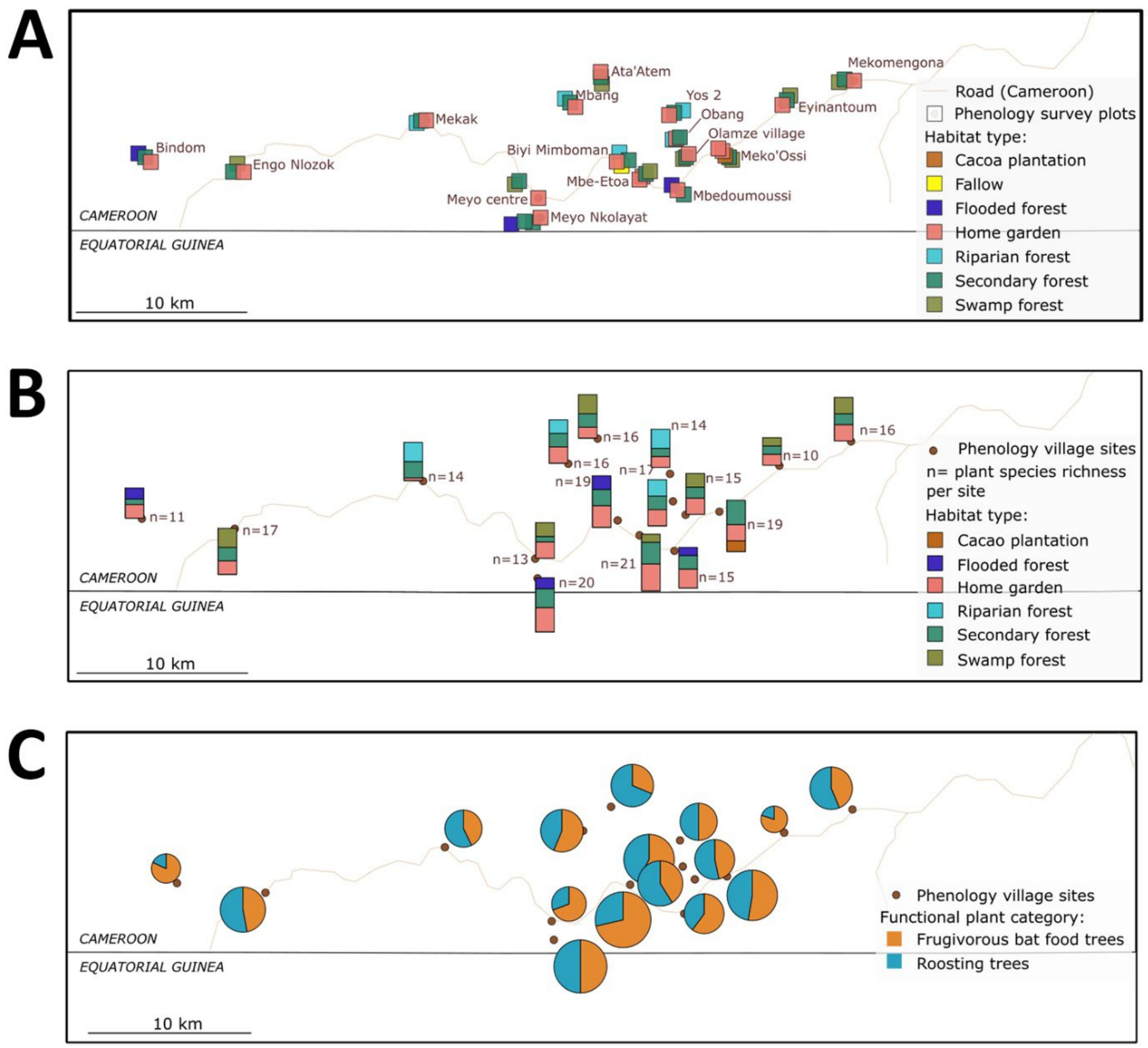

Appendix Figure 4. Plant phenology and habitat characterization at study sites in southern Cameroon. A) Geographic distribution of survey plots across study villages. Each square represents a plot-based inventory location (GPS coordinates). Colors indicate habitat types. B) Species richness and habitat preferences by village. Each point represents one village. Stacked bar plots show the number of species recorded per habitat type; bar height indicates total species richness per village (n). C) Functional classification of recorded plant species. Pie chart shows proportion of food plants (fruit-bearing species known or observed to be consumed by frugivorous bats) versus roosting sites (trees used by bats for shelter).

Appendix Figure 5 (following pages). Questionnaire (In French) given to participants in serosurvey.

FORMULAIRE D'INVESTIGATION DE CAS DE FIÈVRE HÉMORRAGIQUE VIRALE AU CAMEROUN (projet MARCAM)

EPI ID : CMR-FHV-/__/__/__/-/__/__/__/-/__/__/__/__/__/ *Code Région (3) Code district (3) Numéro d'ordre (5)* | **Date de notification :** ______/______/20____/

Fiche remplie par (Noms et prénoms) ____
N° téléphone__________________________ Email______________________________ Fonction____________________
District de santé__________________________ FOSA ______________________ Date d'investigation ______________________
Informations fournies par ☐ Patient ☐ Proche, *si un proche*, Nom du proche ________________ lien de parenté ____________

Section 1 : IDENTIFICATION DU PARTICIPANT

Statut du participant : ☐ Vivant ☐ Décédé si décédé, date de décès /_____/_______/20_____/
Lieu du décès : ☐ Communauté, nom du village/quartier _______________________ District ______________________
☐ Hôpital, nom et service __________________________________ District ______________________
Lieu de l'enterrement, nom du village/quartier ___________________ District ____________________
Noms et prénoms du participant : ____
Fils/fille de (nom père/mère) : ____
Date de naissance : /_____/______/_______/. **Âge** : ____ ☐ Années ☐ Mois **Sexe** : ☐ M ☐ F
Résidence habituelle : Chef de famille (nom et prénom) _______________________ Village/Quartier de résidence : _________
District de santé de résidence : ____________________ **Nationalité** : _______________ **Groupe ethnique** : _____________
Coordonnées GPS domicile : Latitude __________________ Longitude _______________
Coordonnées GPS village : Latitude __________________ Longitude _______________
Profession du participant (cocher la case qui convient) :
☐ Planteur/Paysan ☐ Ménagère/Mère au foyer ☐ Enfant ☐ Chasseur/Marchand de viande de brousse ☐ Médecin traditionnel
☐ Élève/Étudiant ☐ Collecteur de vin de palme ☐ Pêcheur/pêche ☐ Taxi/conducteur/transporteur
☐ Mineur professionnel/artisanal : __________________ date de début des activités minières : __________________
☐ Personnel de santé, Qualification: __________________ structure sanitaire :_________________
☐ Autre, préciser : ____________________________________
Statut marital du participant : ☐ Célibataire ☐ Marié monogame ☐ Marié polygame ☐ Veuf ☐ Divorcé
Antécédents du participant : S'il s'agit d'une femme : ☐ Enceinte ☐ Non enceinte ☐ Ne sais pas
État de santé du participant / maladies sous-jacentes connues par le participant : ______________________________________
Le participant a-t-il voyagé en G.E dans les 3 dernières semaines ou avant le début des symptômes ☐ Oui. ☐ Non
Si oui, préciser (localisation) _________________période (entre) ____/____/____ et ____/____/____
Si oui, préciser raison du voyage : ____
Le participant a-t-il voyagé ailleurs pendant les 3 dernières semaines ou avant le début des symptômes ? ☐ Oui. ☐ Non
Si oui, préciser (localisation) _________________période (entre) ____/____/____ et ____/____/____

Section 2 : Signes et symptômes

Date de début des symptômes /_______/________/20_______/

Fièvre (t°≥38 °C) ☐ Oui ☐ Non ☐ NSP. Si oui, date de début de la fièvre ____/____/____
Est-ce que le participant présente ou a présenté un des symptômes ci-dessous *(cocher les cases correspondantes)*

Céphalées	☐ Oui ☐ Non ☐ NSP	**Saignements**	☐ Oui ☐ Non ☐ NSP
Diarrhée	☐ Oui ☐ Non ☐ NSP	**Si oui :**	
Coliques abdominales	☐ Oui ☐ Non ☐ NSP	Saignements aux points d'injection/prise de sang	☐ Oui ☐ Non ☐ NSP
Vomissements	☐ Oui ☐ Non ☐ NSP	Saignements des gencives (gingivorragie)	☐ Oui ☐ Non ☐ NSP
Fatigue intense	☐ Oui ☐ Non ☐ NSP	Saignements des yeux (injection conjonctivale)	☐ Oui ☐ Non ☐ NSP
Anorexie	☐ Oui ☐ Non ☐ NSP	Selles noirâtres ou avec du sang (mélénas)	☐ Oui ☐ Non ☐ NSP
Douleurs musculaires	☐ Oui ☐ Non ☐ NSP	Sang dans les vomissures (hématémèses)	☐ Oui ☐ Non ☐ NSP
Difficulté d'avaler	☐ Oui ☐ Non ☐ NSP	Vomissement noirâtre (vomito negro)	☐ Oui ☐ Non ☐ NSP
Difficulté de respirer	☐ Oui ☐ Non ☐ NSP	Saignements du nez (épistaxis)	☐ Oui ☐ Non ☐ NSP
Toux intense	☐ Oui ☐ Non ☐ NSP	Saignement vaginal autre que les règles	☐ Oui ☐ Non ☐ NSP
Éruptions cutanées	☐ Oui ☐ Non ☐ NSP	Sang dans les urines (hématurie)	☐ Oui ☐ Non ☐ NSP

Autres signes cliniques non hémorragiques, préciser : ____

Section 3 : Informations sur l'hospitalisation

a. Nom du village dans lequel le patient est tombé malade __________________________ District ____________________
b. Est-ce que le patient s'est déplacé pendant la maladie ☐ Oui ☐ Non
Si oui, remplir la liste indiquant les pays, villages, structures sanitaires :
Pays _______________________Village ______________________Structure sanitaire ____________________________
Pays _______________________Village ______________________Structure sanitaire ____________________________
Pays _______________________Village ______________________Structure sanitaire ____________________________
c. **Transfert du patient à l'hôpital** : Est-ce que le patient a été transféré à l'hôpital ? ☐ Oui ☐ Non

Si oui, Nom de l'hôpital : ___________________________ Date d'hospitalisation /_____/_______/20_____/

Le malade est-il en isolement/en cours d'isolement ? ☐ Oui ☐ Non, Si oui date d'isolement /____/______/20____/

Membre de la famille auprès du malade, Nom et prénom : ____________________________________ **Tel. :** ____________

Le malade était-il hospitalisé ailleurs ou a visité un centre de soins pour la maladie actuelle ? ☐ Oui ☐ Non ☐ NSP

Si oui, veuillez compléter une ligne ci-dessous pour chacune des hospitalisations précédentes :

Dates d'hospitalisation	Nom de la FOSA	Village	District de santé	Le patient était-il en isolement
du _______au ________				☐ Oui ☐ Non
du _______au ________				☐ Oui ☐ Non
du _______au ________				☐ Oui ☐ Non

Section 4 : Épidémiologie/Facteurs de risque

1. Exposition interhumaine (PENDANT LE MOIS PRECEDENT LE DEBUT DES SYMPTOMES)

d. Le participant a-t-il été en contact avec un malade, connu ou suspect, présentant une fièvre, une fatigue, des vomissements sanglants et une diarrhée ? ☐ Oui ☐ Non ☐ NSP

Si oui, veuillez compléter une ligne ci-dessous pour chacun des malades pouvant être une source de contamination

Nom du malade potentiel	Lien de parente	Date(s) du contact	Village	District de santé	Malade vivant ou décédé(e)	Types de contact
					☐ Vivant<br>☐ Décédé, date ___________	
					☐ Vivant<br>☐ Décédé, date ___________	
					☐ Vivant<br>☐ Décédé, date ___________	

Types de contacts (indiquez toutes les possibilités)

1. Contact direct avec les secrétions/excrétions du malade (sang, vomissures, salive, urine, selles)
2. A touché directement le corps du malade (vivant ou décédé)
3. A touché ou partagé linges, habits, plats/assiettes, instruments avec le malade
4. Contact indirect : a mangé avec, a séjourné dans la même maison,
5. Exposition familiale/à l'intérieur du foyer
6. Exposition à l'extérieur du foyer
7. Contact sexuel

e. Le patient a-t-il **visité un proche hospitalisé** **avant** pendant les 3 dernières semaines ou avant le début des symptômes ?
☐ Oui ☐ Non ☐ NSP. *Si oui lieu :* __________________ Date(s) : ____/____/____*et* ____/____/____ *(J,M,A)*

f. Le participant a-t-il été **impliqué dans les soins/transporté un corps d'une personne malade/décédée** pendant les 3 dernières semaines ou avant le début des symptômes ? ☐ Oui ☐ Non
Si oui : Nom et Prénom du malade/défunt : ____

g. Est-ce-que le patient a participé à des funérailles avant la maladie actuelle ? ☐ Oui ☐ Non ☐ NSP

Si oui, veuillez compléter une ligne ci-dessous pour chacune des participations à un enterrement

Nom de la personne décédée	Lien de parente	Date de participations aux funérailles	Village	District de santé	Avez-vous touché le corps ?
		du _________au __________			☐ Oui ☐ Non
		du _________au __________			☐ Oui ☐ Non
		du _________au __________			☐ Oui ☐ Non

h. Le patient a-t-il voyagé en dehors de chez lui ou de son village/ville **avant** la maladie actuelle ? ☐ Oui ☐ Non ☐ NSP
Si oui, Village : ______________________ *District de santé :*_______________ *Date(s) :*___________-___________*(J,M,A)*

i. Le patient a-t-il consulté un **guérisseur/tradipraticien** **avant** la maladie actuelle ? ☐ Oui ☐ Non ☐ NSP
Si oui, Nom : _______________ *Village :*____________ *District de santé* ___________ *Date :*_____/_____/____*(J,M,A)*

j. Le participant a-t-il reçu un **traitement traditionnel** pendant les 3 dernières semaines ou **avant** le début des symptômes ?
☐ Oui. ☐ Non. Si Oui, expliquer quel type de traitement traditionnel : _______________________________

2. Exposition zoonotique

k. Le participant a-t-il été en contact avec un animal sauvage pendant les 3 dernières semaines ou **avant** le début des symptômes ?
☐ Oui ☐ Non ☐ NSP
Si oui, cocher toutes les cases nécessaires

**Animal**	**État** (cocher une case)		**Lieu de contact** (cocher une case)			
☐Chauve-souris	☐ En bonne santé	☐ Malade/mort	☐ Forêt	☐ Plantation/champ	☐ Village	☐ Domicile
☐Singes	☐ En bonne santé	☐ Malade/mort	☐ Forêt	☐ Plantation/champ	☐ Village	☐ Domicile
☐Rongeurs (ou ses excréments)	☐ En bonne santé	☐ Malade/mort	☐ Forêt	☐ Plantation/champ	☐ Village	☐ Domicile
☐Cochons	☐ En bonne santé	☐ Malade/mort	☐ Forêt	☐ Plantation/champ	☐ Village	☐ Domicile
☐Volaille ou oiseaux sauvages	☐ En bonne santé	☐ Malade/mort	☐ Forêt	☐ Plantation/champ	☐ Village	☐ Domicile
☐Vaches, chères ou moutons	☐ En bonne santé	☐ Malade/mort	☐ Forêt	☐ Plantation/champ	☐ Village	☐ Domicile
☐Autres, préciser ___________	☐ En bonne santé	☐ Malade/mort	☐ Forêt	☐ Plantation/champ	☐ Village	☐ Domicile

l. Localisation de l'animal : Pays ______________________ Proximité de quel village : ___________________

m. Le participant a-t-il **visité ou travaillé dans une mine/grotte habitée par des colonies de chauves- souris** pendant les 3 dernières semaines ou **avant** le début des symptômes ? ☐ Oui ☐ Non ☐ NSP
Si oui, Nom de la mine, Localisation ___________________ date _____/_____/_____

n. Consommez-vous les chauves-souris ? ☐ Non ☐ Oui. ☐Si oui, lesquelles ? ☐ les grosses ☐ les petites ☐ les deux

o. Le participant a t'il pratiqué l'une de ces activités lors des 3 dernières semaines ou avant le début des symptômes :

Chasse ou piégeage de chauve-souris	☐ Oui ☐ Non ☐ NSP
Découper de la viande de brousse	☐ Oui ☐ Non ☐ NSP
Vendre de la viande de brousse	☐ Oui ☐ Non ☐ NSP
Trouver un animal mort dans son foyer	☐ Oui ☐ Non ☐ NSP
Consommer un animal mort	☐ Oui ☐ Non ☐ NSP
Être mordu par un animal	☐ Oui ☐ Non ☐ NSP
Consommer des fruits prémâchés par un animal	☐ Oui ☐ Non ☐ NSP
Contact/utilisation de guano de chauve-souris	☐ Oui ☐ Non ☐ NSP
Collecte vin de palme	☐ Oui ☐ Non ☐ NSP
Collecte de fruits	☐ Oui ☐ Non ☐ NSP

p. Consommez-vous régulièrement les produits de la cueillette (fruits) issus de la forêt ? ☐ Oui ☐ Non ☐ NSP
Si oui, préciser les différentes espèces de fruits consommés : ________________________

q. Avez-vous des **arbres fruitiers autour de votre maison ?** ☐ Oui ☐ Non, Si oui, préciser les différentes espèces d'arbres fruitiers
☐ Manguier ; ☐ Avocatier ; ☐ Bananier ;☐ Corossolier ; ☐ Papayer ; ☐ Autres ; A préciser ______________

r. Quelle est **l'heure idéale** à laquelle vous ramassez les fruits tombés des arbres pour la consommation ?
☐ tôt le matin ☐ en matinée ☐ soirée ☐ toute heure de la journée ☐ autre _____________________

s. **Quels sont les arbres fruitiers en pleine fructification dans le village actuellement** ?
☐ Manguier ; ☐ Avocatier ; ☐ Bananier ;☐ Corossolier ; ☐ Papayer ; ☐ Safoutier ; ☐ Autres ; A préciser ________________

t. **Observez-vous régulièrement les chauves-souris dans ces arbres fruitiers ?** ☐ Oui ☐ Non
Si oui, préciser le moment de la journée : ☐ tôt le matin ? ☐ en matinée ? ☐ soirée ?

Section 5 : Prélèvements biologiques pour le laboratoire

Prélèvement ? ☐ Oui ☐ Non ☐ NSP

Prélèvement 1 :
Date de prélèvement : ____/______/_____/
Type de prélèvement
☐ Sang
☐ Urine
☐ Salive
☐ Selles
☐ Biopsie

Prélèvement 2 :
Date de prélèvement : ____/______/_____/
Type de prélèvement
☐ Sang
☐ Urine
☐ Salive
☐ Selles
☐ Biopsie

Résultats			
	Détection d'antigène	☐ pos. ☐ neg ☐ NA	Date ____/_____/_____/
	Sérologie IgM	☐ pos. ☐ neg ☐ NA	Date ____/_____/_____/
	Sérologie IgG	☐ pos. ☐ neg ☐ NA	Date ____/_____/_____/
	RT- PCR	☐ pos. ☐ neg ☐ NA	Date ____/_____/_____/
	Culture du virus	☐ pos. ☐ neg ☐ NA	Date ____/_____/_____/
	Immunohistochimie	☐ pos. ☐ neg ☐ NA	Date ____/_____/_____/
	Immunofluorescence	☐ pos. ☐ neg ☐ NA	Date ____/_____/_____/

Section 6 : Statut final participant

Date à laquelle les informations ont été rapportées : _____/_____/_____
Évolution/Issue (à vérifier 4 semaines après la date de début des symptômes) : ☐ Vivant ☐ Décédé ☐ NSP
Si décédé, date du décès : ______/________/_________
Lieu du décès : ☐Domicile ☐Hôpital ☐Ailleurs, Village/quartier :_______________________District :__________________
Date des funérailles :______/_____/_____ ☐Famille/communauté ☐ Équipe d'enterrement
Lieu des funérailles : Village/quartier :______________________District :_________________

Classification finale du participant *(cocher la case qui convient)*
☐ **Cas Suspect** ☐ **Cas Probable** ☐ **Cas Confirmé** ☐ **Pas un cas** ☐ **Contact**

Version du 24 Juillet 2023
